# Differences in Physical Fitness and Health‐Related Variables Between Older Adults With Versus Without Self‐Reported Impairments in Activities of Daily Living: A Population‐Based Study

**DOI:** 10.1002/ejsc.70210

**Published:** 2026-06-22

**Authors:** Matteo Bergmann, Bettina Barisch‐Fritz, Klaus Bös, Alexander Woll, Janina Krell‐Roesch

**Affiliations:** ^1^ Institute of Sports and Sports Science Karlsruhe Institute of Technology Karlsruhe Germany

**Keywords:** activities of daily living, impairments, objective health, physical complaints, physical fitness, subjective health

## Abstract

Activities of daily living (ADL) are associated with declines in physical fitness and subjective health. However, it remains unclear as to whether ADL impairments are related to specific components of physical fitness and health variables. Therefore, we examined differences between community‐dwelling older persons with versus without ADL impairments with regard to various physical fitness components, physical complaints as well as subjective and objective health outcomes. Cross‐sectional study among 254 participants aged ≥ 55 years [51% female; 84 with ADL impairments; mean (SD) age 62.1 (6.6) years] enrolled in the population‐based “Gesundheit zum Mitmachen” study in Southwestern Germany. ADL, physical complaints and subjective health status were assessed using a self‐report questionnaire, physical fitness (cardiorespiratory fitness, strength, gross motor coordination, flexibility, and functional mobility) was assessed using a fitness test battery, and objective health status was derived from health exam performed by a physician. We ran analyses of covariance, adjusted for age, sex, body mass index and education. Participants with ADL impairments had statistically significantly worse subjective (*p* < 0.001) and objective (*p* < 0.001) health and reported more physical complaints (*p* < 0.001) compared to those without ADL impairments. Regarding physical fitness, ADL‐impaired participants performed worse in 10 out of 12 variables. The findings provide additional evidence that ADL impairments are related to decreased objective and subjective health and physical fitness in older community‐dwelling adults. Future studies employing more comprehensive, preferably objective, ADL assessments and considering cognitive impairments, which may also impact ADL performance, are warranted.

## Highlights

- Community‐dwelling adults aged ≥ 55 years with ADL impairments showed significantly worse subjective and objective health status and had more physical complaints than unimpaired adults.
- ADL‐impaired participants performed significantly worse in 10 of 12 physical fitness variables, spanning cardiorespiratory fitness, strength, gross motor coordination, flexibility, and functional mobility.
- Even minimal ADL impairments were associated with reduced health and fitness, suggesting that early‐stage functional decline may signal broader health deterioration.
- Future research should use longitudinal study design, validated objective ADL measures, account for cognitive status, and explore biological mechanisms behind the observed cross‐sectional associations.

## 1 Introduction

Activities of daily living (ADL) are essential for older adults and reflect a person's functional status or independence (Edemekong et al. 2019). One can distinguish between basic ADL (BADL; such as eating or personal hygiene) and instrumental ADL (IADL; such as doing laundry or shopping). Research has shown that impairments in BADL or IADL is associated with an increased risk of death (Brown et al. 2019), increased care‐related costs (Maresova et al. 2020), an increased risk of cognitive impairments and dementia (Fauth et al. 2013), and reduced quality of life (R. J. Gobbens 2018), amongst others.

Physical fitness refers to the “ability to carry out daily tasks with vigor and alertness, without undue fatigue and with ample energy to enjoy leisure‐time pursuits and to meet unforeseen emergencies” (Caspersen et al. 1985). Components of physical fitness typically include endurance/cardiorespiratory fitness, speed, muscular strength, gross motor coordination, and flexibility (Bös 1987), and distinctions between motor fitness (e.g., agility, strength, speed, and balance) and health‐related fitness (e.g., cardiorespiratory endurance, muscular strength/endurance, body composition, and flexibility) are also common in the literature (Pate 1983).

Physical fitness is critical for health status and well‐being across the lifespan, and particularly for older adults (Bushman and Goddard 2020; Castillo Garzón et al. 2005; Ortega et al. 2018). Physical fitness is also closely related to the ability to perform ADL (Gill et al. 1995), but the longitudinal associations between physical fitness and ADL are less clear. For example, one study showed that higher physical fitness reduced the risk for ADL impairments only in men (Nagamatsu et al. 2003), whereas another showed that only lower gait speed but no other physical fitness variables were predictive of ADL and IADL impairments (R. J. J. Gobbens and van Assen 2014). It has also been reported that a lower baseline performance in functional reach, gait speed, and stair‐climbing ability significantly predicted ADL impairments after 9 years in community‐dwelling older women (Idland et al. 2013). Similarly, low muscle mass, low handgrip strength, low gait speed, and low scores on performance tests have been identified as indicators of worsening ADL (Wang et al. 2020). In contrast, studies also reported no associations between physical test performance and ADL performance, suggesting that the nuances of this association may vary depending on the specific tests used and the populations studied (Clemmensen et al. 2020).

Apart from physical fitness, ADL impairments are also associated with various other measures of health and quality of life. For example, higher self‐rated health is associated with better functional ability and mobility‐related ADL (Gama et al. 2000; Hu et al. 2009), and may predict functional decline even in older adults without baseline disabilities (Fong and Kok 2020).

Despite of a growing body of research on the associations between ADL, physical fitness and health‐related variables, there is still a need for further studies examining these complex relationships. While studies have identified distinct domains (e.g., such as mobility, coordination, fitness, and flexibility) that may explain variances in ADL performance (e.g., (Brach and VanSwearingen 2002)), studies that have used comprehensive, objective assessments of various components of physical fitness are scarce. Also, to the best of our knowledge, no study to date has investigated the associations between ADL with self‐reported physical complaints and physician‐rated health status, or a link between cardiovascular fitness and ADLs. Only one study presented at a conference (Skjødt et al. 2022) showed that between 30% and 10% of community‐dwelling older adults aged 75 years and older use more than 50% of their maximum oxygen uptake for moderate and light ADLs, which could ultimately prove to be a limiting factor in performing these activities.

Therefore, this study aimed to examine differences in physical fitness and health‐related variables between older adults aged ≥ 55 years with versus without self‐reported impairments in ADL. Although the term “older adults” is typically used to refer to individuals aged 65 years and older, we have included individuals as young as aged 55 since research has shown that even in the 50–65 years age group, nearly 15% of adults experience functional impairments and, as a result, have difficulty performing ADLs (Martin and Schoeni 2014; Martin et al. 2010). We hypothesized that individuals with ADL impairments would exhibit lower performance in various physical fitness tests, would report lower subjective health and more physical complaints, and have lower objective health status as rated by a physician. We hypothesize that by leveraging data from the population‐based “Gesundheit zum Mitmachen” study, this study will add to the existing body of research by further providing a more nuanced understanding of the associations between ADL, fitness and health.

## 2 Methods

### 2.1 Design

This cross‐sectional study was conducted in the setting of the population‐based “Gesundheit zum Mitmachen” study in the city of Bad Schönborn in Southwestern Germany (Bös et al. 2012). So far, the study had six measurement waves in 1992, 1997, 2002, 2010, 2015, and 2021. The data for the analysis described in this manuscript was collected in the most recent wave in 2021.

### 2.2 Sample

From a total of 430 participants aged 35–86 years (46% male) who participated in the 2021 measurement wave, 299 adults were aged 55–86 years (49% male). 45 participants had to be excluded due to missing data on variables related to ADL, resulting in a final sample of 254 participants for the current analysis. Study participation was voluntary, and all participants provided written informed consent. Data collection took place during a period of low COVID‐19 prevalence and incidence in Germany, between June 21 and July 29, 2021. The “Gesundheit zum Mitmachen” study was approved by a scientific advisory council, the Schettler Clinic, Bad Schönborn, Germany as well as the ethics committee of the Karlsruhe Institute of Technology.

### 2.3 Measures

A detailed description on the study procedures and assessments has been published previously (Bös and Krell‐Rösch 2022; Woll et al. 2004).

### 2.4 Activities of Daily Living (Independent Variable)

Information on ADL was collected using the FFB‐Mot questionnaire (Woll et al. 2023). The questionnaire contains an ADL scale of four items, that is, for each of the four physical fitness components included in the questionnaire (muscular strength, cardiorespiratory fitness/endurance, flexibility, and gross motor coordination), one item is included in the FFB‐Mot to reflect a low level of difficulty. The items are as follows: sit on a chair and stand up without using your arms (strength), take a brisk walk around several blocks (endurance), put on and take off a narrow sweater and socks by yourself (flexibility), and walk down stairs without grasping the handrail (gross motor coordination). Participants rate each item on a 5‐point Likert scale by indicating as to whether they would be able to carry out the respective activity, with response categories ranging from “I am not able to carry out this activity” (1 point) to “I have no problem carrying out this activity” (5 points). The scores of the FFB‐Mot ADL scale can therefore range between 4 and 20 points, with a higher score reflecting a higher level of fitness. For the current analysis, individuals who scored 20 were categorized as having no ADL limitations, and participants with a score ≤ 19 were categorized as having any ADL limitations.

### 2.5 Subjective Health Status (Dependent Variable)

All participants assessed their health status by responding to the following five questions: “How would you describe your health status?”, “To what extent does your current health status affect your job performance?”, “To what extent does your current health status affect your leisure activities?”, “How would you describe your health status compared to others of your age and gender?” and “Has your health status changed over the past 5 years?” Responses were provided on a 5‐point scale, where 1 represented very poor health and 5 indicated excellent health. An overall self‐rated health status score was computed, ranging from 5 to 25 points, with higher scores reflecting better perceived health status. The questions to assess subjective health status were derived and modified from validated instruments (Antonovsky 1979, 1987; Becker 1992, 1994).

### 2.6 Objective Health Status (Dependent Variable)

Objective health status in three domains, that is, orthopedics, neurology, and cardiovascular system was derived from a comprehensive health examination and medical history (anamnesis) conducted by a physician. Each domain was scored as either 0 (no limitations), 1 (minor limitations, not affecting daily life), 2 (limitations affecting daily life), and 3 (major limitations severely affecting daily life). A composite physician‐rated health status score ranging from 0 to 9 was calculated by summing the scores across the three domains, with a higher score reflecting worse objective health status.

### 2.7 Physical Complaints (Dependent Variable)

Subjective impairments due to psychosomatic complaints (e.g., feelings of weakness, chest pain) were assessed using the self‐administered B‐LR List of Complaints (Zerssen Complaint List), which measures the severity of symptoms on a scale ranging from no symptoms to severe impairments, yielding a sum‐index score from 0 to 72 with higher values reflecting higher complaints (von Zerssen and Petermann 2011). The reliability of the scale is high, with a split‐half reliability coefficient of *r* = 0.93.

### 2.8 Physical Fitness (Dependent Variable)

A motor performance test battery was conducted to assess physical fitness. All tests were performed in a single session and were supervised by trained staff according to a standardized protocol (Suni et al. 1996). Following the health examination by the physician, participants completed tests to assess strength, gross motor coordination, flexibility, and functional mobility in a flexible sequence, depending on test station availability. Cardiorespiratory fitness was assessed last for all participants. For this purpose, the time to complete a 2‐km walking test (in minutes and seconds, lower value reflects better cardiorespiratory fitness) (Oja et al. 1991) was measured. Strength was assessed with a handgrip test performed twice with both hands (digital hand dynamometer, 198 lbs, GRIPX, China; measuring kilograms, higher value reflects higher strength; highest value of trials from left and right hand used for analysis) and a jump‐and‐reach test (in cm, higher value reflects higher strength). Gross motor coordination was measured through five tasks: (1) Single‐leg stand: Standing on one leg with eyes closed while moving the other leg in circles; (2) Wall throw: throwing a ball against a wall and catching it, (3) Throw and turn: Throwing a ball with rotation under time pressure and catching it, (4) Gripping a ball: Holding a ball between the legs with one hand in front of one thigh and the other hand behind the opposite thigh, then releasing and catching the ball while alternating grips five times; (5) Circling eights: Standing next to two cones, describing five figure‐eight pattern by making circles around two cones with one leg in the air. Each gross motor coordination task was rated as 2 (task completed easily), 1 (task completed with difficulty), 0 (task not completed), and a coordination index (possible range: 0–10 points; higher value reflecting better coordination performance). A total score for gross motor coordination was calculated by summing up the five test items. Flexibility was measured using the sit‐and‐reach test and a trunk side‐bending test (in cm, higher value reflects higher flexibility). Additionally, a functional mobility index (possible range: 0–12 points, higher value reflects higher functional mobility) was calculated based on six three‐point‐scaled items assessing the extensibility of the shoulder‐neck (left/right), hamstrings (left/right), and rectus femoris (left/right) muscles (2, no restrictions; 1, moderate restrictions; 0, major restrictions). For the jump‐and‐reach, sit‐and‐reach, and gross motor coordination tasks, the best result of two trials was used in the analyses. All other tasks were performed once. The test battery was developed in collaboration with the UKK Institute in Tampere, Finland (Suni et al. 1996). Means and standard deviations of all physical fitness test values are reported in the results section.

### 2.9 Additional Measures

We included age, sex, education and body mass index (BMI) as covariates in the analyses. BMI was calculated using the formula kg/m^2^. Body weight was measured using a calibrated scale (Seca GmbH & Co. KG, Hamburg, Germany) on a solid surface, and body height using a mobile stadiometer. Socioeconomic status was calculated based on information about educational level and occupational group affiliation, and four categories were created for data analysis (i.e., low, mid/low, mid/high, and high) (Hradil 1987).

### 2.10 Statistical Methods

Descriptive statistics are presented for categorical variables through number (*N*) and percentage (%), and for continuous variables through means and standard deviations (SD). Multicollinearity was tested by calculating the variance inflation factor (VIF) for all variables, and all VIF values were below 1.1 indicating no relevant multicollinearity. Differences in characteristics such as education and socioeconomic status between participants with versus without ADL impairments were analyzed using Chi‐square test. Differences in the dependent variables (physical fitness, subjective and objective health status, and physical complaints) between participants with versus without ADL impairments were examined using analysis of covariance (ANCOVA). We ran models adjusted for age, sex, and BMI (model 1), and additionally adjusted for education (model 2). The categorical variable education was transformed into a binary dummy variable prior to statistical analysis. Partial eta‐squared were calculated as measures of effect size. Furthermore, mean differences and 95% confidence intervals were computed for the pairwise comparisons to provide an estimate of the precision of the observed effects. Analyses were performed using SPSS Statistics 30 for Windows (IBM, Armonk, New York), with level of significance set at *p* < 0.05.

## 3 Results

Of 254 participants, we classified 170 as having no ADL impairments (87 female, 83 male), and 84 as having ADL impairments (42 female, 42 male). Mean (SD) age was 62.1 (6.6) years [ADL‐unimpaired: 61.7 (6.0); ADL‐impaired: 62.9 (7.6)]. Mean (SD) BMI was 25.7 (4.1) [ADL‐unimpaired: 25.5 (4.0); ADL‐impaired: 26.1 (4.1)]. A significant association was found between ADL‐impaired and unimpaired participants for education (*p* = 0.03), but not socioeconomic status (*p* = 0.17). Please refer to Table 1 for an overview of participants' characteristics.

**TABLE 1:** Characteristics of participants.

	Total *N* = 254	ADL‐unimpaired *N* = 170	ADL‐impaired *N* = 84
Age in years, mean (SD)	62.1 (6.6)	61.7 (6.0)	62.9 (7.6)
Female sex, *N* (%)	129 (51)	87 (51)	42 (50)
BMI, mean (SD)	25.7 (4.1)	25.5 (4.0)	26.1 (4.1)
ADL‐score (19), *N* (%)			44 (52)
ADL‐score (18), *N* (%)			16 (19)
ADL‐score (17), *N* (%)			6 (7)
ADL‐score (16), *N* (%)			5 (6)
ADL‐score (15), *N* (%)			2 (2)
ADL‐score (≤ 14), *N* (%)			11 (13)
Education			
No degree, *N* (%)	2 (1)	0 (0)	2 (2)
Secondary general school, *N* (%)	48 (20)	28 (17)	20 (24)
Vocational school, *N* (%)	79 (32)	55 (32)	24 (29)
University entrance qualification, *N* (%)	86 (35)	58 (34)	28 (33)
University degree, *N* (%)	30 (12)	22 (13)	8 (10)
Socioeconomic status			
Low, *N* (%)	38 (15)	20 (12)	18 (21)
Mid/low, *N* (%)	37 (15)	24 (14)	13 (16)
Mid/high, *N* (%)	133 (53)	91 (54)	42 (50)
High, *N* (%)	44 (18)	33 (20)	11 (13)

*Note:* Higher score in ADL‐score reflects lower impairments.

Abbreviations: ADL, activities of daily living; BMI, body mass index.

After adjusting for age, sex, and BMI, participants with ADL impairments as compared to those without had statistically significantly worse subjective health status (*p* < 0.001), and objective health status (*p* < 0.001) as well as more physical complaints (*p* < 0.001). With regard to physical fitness variables, participants with ADL impairments performed worse than those without impairments in 10 of 12 variables, that is, cardiorespiratory fitness (*p* < 0.001), gross motor coordination [total score (*p* < 0.001), circling eights (*p* < 0.001), wall throw (*p* = 0.02), throw and turn (*p* = 0.03), gripping a ball (*p* = 0.002)], strength [handgrip strength (*p* = 0.03), jump and reach (*p* = 0.003)], functional mobility (*p* = 0.002), and flexibility [sit and reach (*p* = 0.03)]. Additional adjustments of the models for education did not alter the results. Please refer to Table 2 for the results from ANCOVA.

**TABLE 2:** Results of ANCOVA for differences between ADL‐unimpaired and ‐impaired participants on health‐related and fitness‐related variables.

Variable	*N*	ADL‐unimpaired		ADL‐impaired		Model 1				Model 2			
		*N*	Mean (SD)	*N*	Mean (SD)	*F*	Sig.	Part. *η* ^2^	Mean difference (95% CI)	*F*	Sig.	Part. *η* ^2^	Mean difference (95% CI)
Health‐related variables													
Subjective health status	250	166	17.52 (3.16)	84	14.76 (3.58)	36.010	**<** **0.001**	0.131	2.749 (1.847–3.652)	37.772	**<** **0.001**	0.142	2.769 (1.881–3.656)
Objective health status	244	163	0.43 (0.8)	61	1.01 (1.27)	17.861	**<** **0.001**	0.071	−0.572 (−0.839–−0.306)	19.643	**<** **0.001**	0.081	−0.542 (−0.783–−0.301)
Zerssen complaint list	215	151	12.87 (8.31)	64	20.00 (9.58)	26.104	**<** **0.001**	0.113	−6.741 (−9.342–−4.140	22.239	**<** **0.001**	0.101	−6.382 (−9.050–−3.713)
Fitness‐related variables													
Cardiorespiratory fitness	224	153	17.38 (1.95)	71	18.97 (2.86)	25.284	**<** **0.001**	0.106	−1.659 (−2.309–−1.009)	25.684	**<** **0.001**	0.112	−1.681 (−2.335–−1.027)
Coordination (total score)	239	161	7.81 (4.67)	78	5.09 (4.23)	20.538	**<** **0.001**	0.083	2.814 (1.590–4.037)	22.561	**<** **0.001**	0.094	2.944 (1.723–4.166)
Coordination subtests													
Circling eights	236	161	1.58 (0.69)	75	1.17 (0.89)	15.038	**<** **0.001**	0.063	0.424 (0.208–0.639)	14.240	**<** **0.001**	0.062	0.419 (0.200–0.639)
Wall throw	238	160	0.86 (0.87)	78	0.63 (0.81)	6.052	**0.02**	0.026	0.283 (0.056–0.511)	6.336	**0.01**	0.028	0.298 (0.065–0.532)
Single leg stand	236	161	0.52 (0.76)	75	0.33 (0.66)	2.618	0.11	0.012	0.167 (−0.036–0.371)	3.177	0.08	0.014	0.186 (−0.020–0.393)
Throw and turn	237	159	0.93 (0.80)	78	0.69 (0.71)	5.090	**0.03**	0.022	0.237 (0.030–0.445)	5.567	**0.02**	0.025	(0.253 (0.042–0.465)
Gripping a ball	237	159	1.23 (0.83)	78	0.88 (0.87)	10.036	**0.002**	0.043	0.358 (0.135–0.580)	9.426	**0.002**	0.042	0.352 (0.126–0.577)
Strength subtests													
Handgrip strength	229	155	36.41 (12.42)	74	33.89 (11.87)	4.973	**0.03**	0.022	2.626 (0.305–4.948)	5.080	**0.03**	0.024	2.767 (0.347–5.188)
Jump and reach	227	156	25.22 (8.80)	69	22.26 (8.57)	9.290	**0.003**	0.041	3.342 (1.181–5.504)	7.937	**0.005**	0.037	3.086 (0.926–5.246)
Functional mobility (total score)	238	161	8.53 (2.86)	77	7.29 (2.91)	10.300	**0.002**	0.043	1.215 (0.469–1.961)	12.328	**<** **0.001**	0.054	1.366 (0.599–2.133)
Flexibility subtests													
Sidebend	239	161	20.84 (4.68)	78	19.74 (5.01)	3.171	0.08	0.014	1.161 (−0.124–2.445)	3.639	0.06	0.016	1.248 (−0.041–2.537)
Sit and reach	237	160	3.60 (10.46)	77	0.44 (10.80)	5.060	**0.03**	0.022	3.010 (0.373–5.647)	5.325	**0.02**	0.024	3.194 (0.466–5.922)

*Note:* Higher score reflects better performance/status for subjective health status, gross motor coordination total score and subtests, strength subtests, functional mobility total score, flexibility subtests; lower score reflects better performance/status for objective health status, Zerssen complaint list, cardiorespiratory fitness; Model 1 adjusted for age, sex, and BMI; model 2 additionally adjusted for education; *p*‐value bold when statistically significant (*p* < 0.05).

Abbreviations: ADL, activities of daily living; CI, confidence interval.

## 4 Discussion

We observed that community‐dwelling adults aged ≥ 55 years with ADL impairments have lower subjective and objective health and reported more physical complaints compared to those without ADL impairments. Also, in line with our hypotheses, participants with compared to those without ADL impairments performed worse in 10 out of 12 physical fitness variables including cardiorespiratory fitness, strength, gross motor coordination, flexibility and functional mobility.

However, no statistically significant differences were observed for performance in the side bend and single‐leg stand tests. The reasons for this remain unclear, as previous literature found that single‐leg stand is a valid and reliable measure of balance (Frändin et al. 1995), and that balance is related to ADL impairments (Judge et al. 1996). A similar relationship between educational level and selective cognitive abilities was also observed by Zamarian and colleagues (Zamarian et al. 2021), which was also related to ADL performance in their study.

In line with a growing body of existing research, our present findings confirm the predominantly established relationship between ADL and physical fitness (Gill et al. 1995; Judge et al. 1996). Albeit, few studies also exist that did not find an association between physical performance tests and ADL performance (Clemmensen et al. 2020). This lack of an association could be attributed to the selection of specific tests, and in our study, we also had two physical fitness tests for which performance did not differ between ADL‐impaired and unimpaired participants. Additionally, there are differences between studies with regard to the sample size, for example, Clemmensen and colleagues (Clemmensen et al. 2020) included participants with mild to moderate Alzheimer's disease, and in this population, one may speculate that ADL impairments may primarily be attributable to cognitive impairments.

In our study, we also found that ADL‐impaired compared to unimpaired persons had lower self‐rated health status, with is in concordance with prior studies such as those by Gama and colleagues (Gama et al. 2000) and Hu et al. (2009), which also reported associations between self‐rated health and ADL. To the best of our knowledge, our study may be among the first to show that ADL impairments is also related to lower objective health status and higher physical complaints.

Strength of our study are the rigorous assessment of physical fitness using a comprehensive, 13‐item test battery, and assessment of subjective health status corroborated by a health exam carried out by a physician.

A main limitation of this study is the cut‐off point used to distinguish between ADL‐impaired and ADL‐unimpaired persons. Even a minimal self‐reported impairment in one of the four different ADL items was sufficient to classify someone as having ADL impairments. As evident in Table 1, the majority of ADL‐impaired participants have only mild ADL impairments. However, since we observed significant differences between ADL‐impaired versus unimpaired participants for physical fitness and health‐related variables, our study provides preliminary evidence that even minimal ADL impairments in community‐dwelling older adults may be associated with decreased health and physical fitness. One may anticipate that the differences between ADL‐impaired and unimpaired persons would become even more pronounced in a sample with more severe impairments in ADL. Moreover, it must be noted that the assessment of ADL performance was not very comprehensive and was only based on four questions. A more holistic approach using valid, preferably more objective ADL assessments would be desirable, potentially considering different subdomains of ADL. Furthermore, participants completed the physical fitness tests in a flexible sequence, except for the 2‐km walking test which was consistently performed last; thus, a potential influence of fatigue cannot be ruled out. Another main limitation of our study pertains to the cross‐sectional design which does not allow for drawing any conclusion about causality in the associations between ADL impairments with physical fitness, physical complaints or health status. Thus, future studies in more diverse and heterogeneous populations with regard to ADL impairments are warranted to investigate these complex relationships, preferably also using validated and reliable objective ADL assessments, and also taking into account cognitive status of older adults, which may have an impact on ADL performance (Clemmensen et al. 2020; Romero‐Ayuso et al. 2021). Also, research on the biological mechanisms underlying the associations between ADL impairments and performance in certain physical fitness tests is required, for example, it is unknown as to why we did not observe a difference between ADL‐impaired and unimpaired participants in single leg stand and side bend test, but observed a difference in all other 10 fitness variables.

## 5 Conclusion

Our study provides further evidence of an association between ADL with subjective and objective health status, physical complaints and physical fitness in community‐dwelling adults aged ≥ 55 years. This finding has important implications for clinical practice, policy makers and stakeholders, as it may emphasize the importance of targeted (lifestyle) interventions to counteract impairments in ADL, thereby potentially also promoting and maintaining physical fitness and health in older adults.

**Abbreviations**
ADL: Activities of daily living
BADL: Basic activities of daily living
BMI: Body mass index
CI: Confidence interval
e.g.: Example given
FFB‐Mot: Funktionsfragebogen Motorik (engl. functional fitness questionnaire)
IADL: Instrumental activities of daily living
M: Mean
N: Number
SD: Standard deviation
VIF: Variance Inflation Factor

## Author Contributions

All authors contributed to the study conception and design. Material preparation, and data collection were performed by J.K.‐R., A.W. and K.B., and data analysis was performed by MB. The first draft of the manuscript was written by M.B., B.B.‐F. and J.K.‐R., and all authors commented on previous versions of the manuscript. All authors have read and approved the final manuscript.

## Funding

The authors acknowledge funding from AOK Mittlerer Oberrhein and the city of Bad Schönborn for this research. Open Access funding enabled and organized by KIT‐Publication Fund of the Karlsruhe Institute of Technology. Matteo Bergmann is supported by a scholarship of the Baden‐Württemberg Landesgraduiertenförderung (LGF) program.

## Ethics Statement

The study and all procedures performed in this research were approved and carried out in accordance with the ethical standards of the ethics committee of the Karlsruhe Institute of Technology, Germany. Written informed consent was obtained from all individuals participating in the study.

## Consent

The authors have nothing to report.

## Conflicts of Interest

The authors declare no conflicts of interest.

## Data Availability

The data that support the findings of this study are available from the corresponding author upon reasonable request.
